# Chemokine/GPCR Signaling-Mediated EMT in Cancer Metastasis

**DOI:** 10.1155/2022/2208176

**Published:** 2022-10-11

**Authors:** Xutengyue Tian, Jiayi Wang, Lanxin Jiang, Yuchen Jiang, Juan Xu, Xiaodong Feng

**Affiliations:** ^1^State Key Laboratory of Oral Diseases, National Clinical Research Center for Oral Diseases, Research Unit of Oral Carcinogenesis and Management, Chinese Academy of Medical Sciences, West China Hospital of Stomatology, Sichuan University, Chengdu, Sichuan 610041, China; ^2^Department of Stomatology, Sijing Hospital, Shanghai 210061, China

## Abstract

Metastasis, the chief cause of cancer-related deaths, is associated with epithelial-mesenchymal transition (EMT). In the tumor microenvironment, EMT can be triggered by chemokine/G-protein-coupled receptor (GPCR) signaling, which is closely associated with tumor progression. However, the functional links between chemokine/GPCR signaling-mediated EMT and metastasis remain unclear. Herein, we summarized the mechanisms of chemokine/GPCR signaling-mediated EMT with an insight into facilitating metastasis and clarified the role of chemokine in the local invasion, intravasation, circulation, extravasation, and colonization, respectively. Moreover, several potential pathways that might contribute to EMT based on the latest studies on GPCR signaling were proposed, including signaling mediated by G protein, *β*-arrestin, intracellular, dimerization activation, and transactivation. However, there is still limited evidence to support the EMT programme functional contribution to metastasis, which keeps a key question still open whether we should target EMT programme of cancer cells. Answers to that question might help develop an anticancer strategy or guide new directions for anticancer metastasis therapy.

## 1. Introduction

Chemokines are a group of small (8∼14 kDa) structurally related molecules that participate mainly in leukocyte trafficking via GPCR signaling. Approximately 48 ligands are found to bind to 19 different chemokine receptors nowadays[1]. And most chemokine receptors are G*α*i-coupled GPCRs regulating directed cell migration, adhesion, and cell-cell interactions. In the tumor microenvironment, chemokines are major drivers of tumor progression, angiogenesis, metastasis, and even EMT in multiple cancers.

EMT was first proposed by Elizabeth Hay in the field of development, where cells undergoing EMT remodel their cytoskeletons, gain front-back polarity, and abandon tight junctions [2]. Interestingly, in neoplasms, a complete-EMT (c-EMT) is rare; instead, it is often “hijacked” partly by malignant cells, termed the partial-EMT (p-EMT). Those programmes were recognized as important in the context of cancer, owing to their potential in conferring malignant cells with motility, stemness, and invasiveness, which allows malignant cells to find a new place to colonize [3].

Metastasis is the chief cause of cancer-related death (about 90% [4]). However, metastatic dissemination is not efficient; once cancer cells leave their favorable surroundings, they are exposed to various threats such as shear stress, which may lead to death [5]. Only a few cancer cells succeed in forming metastases, and they must complete metastatic processes, including local invasion, intravasation, transportation through circulation, extravasation, and colonization. They have the traits to exploit the microenvironment, transform themselves for adaptation, survive, and form distant metastases [6, 7]. Currently, one of the key phenotypes discovered in cancer cells throughout metastatic dissemination is the mesenchymal phenotype, regarded as a consequence of EMT [8]. However, whether chemokine/GPCR mediates metastasis through EMT programmes or should we focus on targeting EMT programmes for anticancer metastasis therapies development remains unclear.

## 2. EMT and Metastasis in Brief

EMT is a cellular programme in which epithelial cells acquire mesenchymal phenotypes with a loss of epithelial phenotypes [2]. This programme is known to be associated with tumor initiation, invasion, migration, stemness, proliferation, and chemoresistance and has received extraordinary attention for EMT-associated protein markers for their clinical significance in evaluating the malignancy of cancer. In the tumor microenvironment, cancer cells tend to undergo a partial-EMT (p-EMT) programme instead of a complete-EMT (c-EMT) programme [3]. Unlike the c-EMT programme, the p-EMT programme is used to describe cancer cells in an epithelial-mesenchymal intermediate state, manifesting as simultaneously expressing both epithelial and mesenchymal markers or simply loss of epithelial markers with no change in mesenchymal markers [9]. To date, the EMT programme is believed to be triggered by the stroma in the malignant tissue, which is mainly composed of carcinoma-associated fibroblasts (CAFs), tumor-associated macrophages (TAMs), T lymphocytes, and myeloid-derived suppressor cells [10]. Together, they employ paracrine or direct contacts to induce EMT in carcinoma cells, which transduce signaling ending up at EMT-TFs (Snail, Slug, Twist1/2, and Zeb1/2) [10].

EMT programmes have been reported to be associated with multiple steps. In primary tumor, EMT cells are reported to localize at the leading edge of the tumor, which is in proximity to stroma [11, 12], exhibiting invasive features that secrete matrix metalloproteases (MMPs) and form invadopodia [13, 14] to remodel ECM. Twist has been reported specifically enhanced in intravasation [14, 15]. Circulating tumor cells (CTCs) have been reported to have mesenchymal characteristics and express EMT regulators, such as components of the TGF-*β* pathway and FOXC1 [16]. Notably, the source of TGF-*β* in circulation is likely to be platelets that attach to CTCs and help these cells survive in circulation [16, 17]. Attachment of cancer cells to endothelial cells is the first step in extravasation.And N-cadherin, a marker of mesenchymal cells, expressed both by endothelial and some cancer cells, is important for attachment and extravasation [18]. Interestingly, even the relationship between EMT and metastasis was carefully reviewed by Mittal [11], and most studies successfully established the association of them, but still, very limited concrete experimental evidence has proven the EMT programme a potent regulator of cancer cell dissociation from the primary tumor and crossing the endothelial barrier.

Clinically, the EMT-TFs have been found critical prognostic value in head and neck squamous cell carcinoma (HNSCC) [19]. Overexpression of Twist1, Snail1, Snail2, and ZEB1 indicated a poor overall survival (OS) of HNSCC [19]. And EMT status of circulating tumor cells (CTCs) is also associated with HNSCC recurrence [20]. Single-cell transcriptomic analysis revealed that head and neck squamous cells mainly undergo the p-EMT programme instead of the c-EMT programme. Notably, among the classical EMT-TFs, only Snail2 was detected, hinting at a post-transcriptional regulation [12]. Immunohistochemistry stained these p-EMT cancer cells at the leading edge of the tumor, closely interacting with CAFs [12]. And, flow cytometry has confirmed the significance abundance of CAFs in human HNSCC [21]. These studies indicated the possible critical role of CAFs in mediating EMT in HNSCC. However, the influence of TAMs cannot be excluded even though there are limited studies.

## 3. Chemokine-Mediated EMT and Associated Metastasis

**Figure 1:**
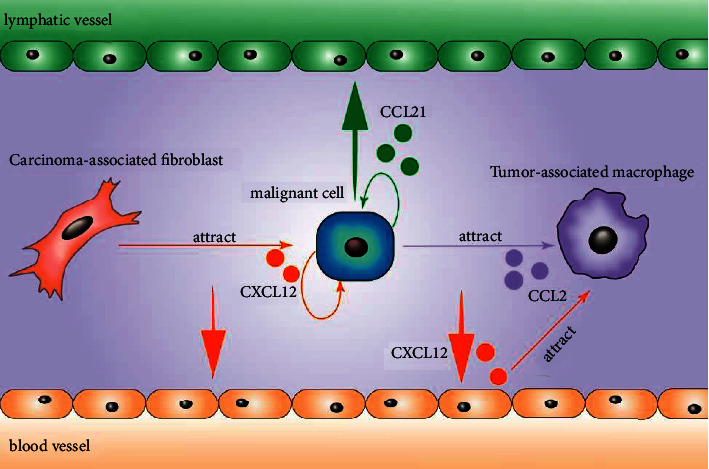
Interaction between cancer cells and non-cancer cells via chemokine. CXCL12 is secreted by CAFs, malignant cells, and endothelial cells. Malignant cells migrate along CXCL12 gradient to blood vessels with the help of CAFs or independently. CCL2 is secreted by malignant cells to recruit TAMs. They migrate toward blood vessels with the help of TAMs. CCL21 gradient directs malignant cells to lymphatic vessels via autocrine way. CAFs, carcinoma-associated fibroblasts and TAMs, tumor-associated macrophages.

The chemokine superfamily comprises a large number of ligands and receptors. In further studies, the confusing ligand-receptor relationships were gradually unveiled and categorized into two subgroups: “inflammatory” and “homeostatic”. A plethora of literature has shown that chemokines from both subgroups are able to induce EMT (see Table 1) and promote metastasis. In addition to mediating EMT, chemokines also contribute to directed migration and transendothelial migration of cancer cells and recruitment of non-cancer cells in the whole dissemination process (Figure 1).

  CXCL12-CXCR4 axis has been well studied in cancer, with a significant effect on mediating EMT in a variety of cancers, such as gastric cancer, breast cancer, head and neck cancer, and lung cancer (see Table 1). In the context of metastasis, CXCL12 as a typical homeostatic chemokine regulates directional metastasis, which might be a result of its function in mediating EMT, directing cell migration. Signaling through CXCL12-CXCR4 could mediate intracellular actin polymerization and induce typical pseudopodia formation after 20 minutes of stimulation, suggesting an active mesenchymal transition [83]. MCF-7 cells stably expressing SDF-1 (CXCL12) observed significant downregulation of E-cadherin and enhanced expression of Slug, fibronectin, and vimentin through the NF-*κ*B pathway [84]. In a 2D environment, MCF-7 expressing constitutively active CXCR4 exhibited upregulation of ZEB1 and cadherin 11, loss of E-cadherin, and p120 isoform switching [32]. Interestingly, MCF-7 CXCR4 WT cells displayed epithelial characteristics under 2D condition but mesenchymal characteristics under 3D culture. This structural discrepancy between 3D and 2D cultures indicates that confinement may be critical for regulating EMT [32].
  CXCL12 is mostly recognized to be secreted by CAF in a paracrine way [85]. Accordingly, cancer cells upregulate the expression of CXCR4 with increased vimentin and decreased E-cadherin upon sensing CAF-produced CXCL12 in a coculture system [86]. These CXCR4-positive cells thus migrate directionally along the CXCL12 gradient toward their destination, such as blood vessels [85, 87]. Additionally, CXCR4-positive tumor cells might exploit interstitial fluid to form autologous gradients that direct them to blood vessels [88, 89].
  The role of CXCL12 in effective vascular barrier crossing and distal colonization has been extensively studied. For example, CXCL12 can directly promote the adhesion of cancer cells to endothelial cells or induce morphological changes via GTPase-mediated signaling [90, 91]. After extravasation to metastasized tissues, the main problem is how to adapt and thrive in new microenvironments. Zhang et al. proposed a view that stromal signals resembling those of a distant organ select for cancer cells that are primed for metastasis in that organ. For example, triple-negative breast tumor cells that tend to thrive on the CAFs-derived CXCL12 and IGF are primed for metastasis in the CXCL12-rich microenvironment [92].
  CCL2-CCR2 axis not only acts on cancer cells to mediate EMT and transendothelial migration but also recruits TAMs to assist tumor progression and metastasis. CCL2 is an inflammatory chemokine that attracts monocytes, natural killer cells, and memory T cells to mediate multiple inflammatory processes. In neoplasms, CCL2 is well recognized to have a promalignant effect with TAMs. A recent study indicates that CCL2 attracts circulating monocytes into the tumor and triggers their acquisition of a TAM phenotype [93]. In turn, the TAMs display a tumor-promoting effect in the tumor microenvironment. A recent study indicated that ZEB1 expression is required for the activation of macrophages toward a protumor TAM phenotype, which enhances the expression of CCR2 and MMP9. Through MMP9, TAMs activate CCL2 expression in cancer cells. Together they form a CCR2-MMP9-CCL2 positive loop that drives tumor progression and are probably critical in triggering the EMT programme [94]. In addition to cancer cells, CCL2 is also secreted by TAMs. In colorectal cancer, TAMs secrete CCL2 via Wnt5a/CaMKII/ERK/CCL2 axis, and those CCL2 molecules act on cancer cells and drive tumor progression [95].
  CCL2-CCR2 axis has been reported to mediate EMT in various cancers, including hepatocellular, gastric, prostate, breast, head, and neck cancer (Table 1). Like CXCL12, most researchers incubated carcinoma cells, which have been starved overnight, with CCL2 for about 48 h, and witnessed alterations in cell morphology and EMT markers. For example, in breast cancer, MCF-7 incubated with CCL2 (50 ng/ml) observed spindle morphology and increased Snail, E-cadherin, and vimentin expression. The MEK1/2 and GSK-3 beta could reverse the CCL2-induced EMT phenotype, indicating that MAPK/GSK-3-beta/Snail signaling is involved in EMT regulation [60]. Interestingly, no apparent changes in Snail mRNA levels were shown during CCL2 treatment. This might be a result of post-transcriptional regulation of CCL2.
  Study about CCL2 in metastatic dissemination is limited. Current studies have reported CCL2 attracting monocytes to the primary tumor that facilitate migration. These monocytes subsequently differentiate into migratory TAMs that carry cancer cells along the CXCL12 gradient secreted by perivascular fibroblasts toward blood vessels [96]. In further process, the CCL2 secreted by cancer cells can also interact with CCR2 on endothelial cells to facilitate the transendothelial process [6].
  CCL19/CCL21-CCR7 axis also plays a vital role in EMT (see Table 1) and lymphatic metastasis. CCL19 and CCL21, two known ligands of CCR7, display nonoverlapping roles in the homing of the immune system, including trafficking of T cells and dendritic cells [97]. For example, CCL19 functions in the thymic emigration of mature T cells from fetal thymic organ cultures, but not CCL21 [98]. Therefore, even if they share the same receptor, their roles in EMT or tumor progression might be slightly different. Cancer cells incubated with CCL19 (50 ng/ml) or CCL21 (100 ng/ml) showed increased expression of N-cadherin and vimentin and decreased expression of E-cadherin. In this process, adverse signaling pathways were involved such as PI3K/Akt, JAK/STAT, and MAPK pathways.
  The specific origin of CCL19 and CCL21 in the tumor microenvironment remains unclear. Studies have found CCL21 expression in the high endothelial venules of lymph nodes, which connects it with lymphatic metastasis. For now, several studies have found a correlation between CCR7 expression and lymph node metastasis [99, 100].
  Studies on the mechanism of which CCL19/CCL21 attracts cancer cells toward lymph nodes are unclear. A study proposed that CCL21/CCL19-CCR7 axis mediated the directional migration of cancer cells to lymphatics with an autocrine fashion. Specifically, CCL21/CCL19 secreted by tumor cells with membrane CCR7 generates an autologous gradient, along which tumor cells migrate to lymphatics [89]. Additionally, CCL21 secreted by lymphatic can also guide lymphatic metastasis [101, 102].
  The deregulation of chemokines has been observed in HNSCC via bioinformatics analysis of The Cancer Genome Atlas (TCGA). Chemokines such as CXCL1, CXCL8, CXCL13, CCL3, and CCL5 are reported upregulation in mRNA levels [103]. Interestingly, the well-studied chemokines such as CXCL12, CCL2, and CCL7 were not obviously deregulated in mRNA levels, indicating a post-transcriptional modification. CXCL12 is well studied in HNSCC and has prognostic value. Patients with higher expression of CXCL12/CXCR4 have higher risk of regional recurrence [104]. CXCL12 secreted by CAFs or malignant cells can activate the EMT programme via the PI3K-Akt pathway (Table 1) and promote invasion via MAPK-AP1 and NF-*κ*b pathways [105, 106]. CCL2 can be secreted by CAFs and malignant cells to assist tumor progression, EMT, and migration [107]. CCL2-CCR4 axis, not the CCL2-CCR2 axis, is reported to facilitate cell migration via Vav guanine nucleotide exchange factor 2 (Vav2) Rac family small GTPase1 (Rac1) complex-myosin light chain (MLC) signaling [108]. However, we still have few clues on how CCL2 contributes to EMT in HNSCC. CCR7 in HNSCC is associated with lymph node metastasis [109]. CCL19-CCR7 axis facilitates migration, invasion, and adhesion with the involvement of PI3K-cdc42, MMP9, RhoA/ROCK, and *β*-1 integrin [110–113], while JAK-STATs and MAPK pathways have been reported correlated with EMT activation (Table 1).

Above all, these studies reflect the complexity of chemokine/GPCR-mediated EMT-associated metastatic dissemination of cancer cells, in which cancer cells, non-cancer cells, cytokines, and the ECM should be considered. Further studies are required to clarify how these factors work together to promote metastasis. Only a few studies demonstrated morphological changes among cells under microscopes, perhaps because the p-EMT programme rather than the c-EMT programme is more common in cancer.

## 4. Signaling Pathways Inducing EMT by Chemokines

**Figure 2:**
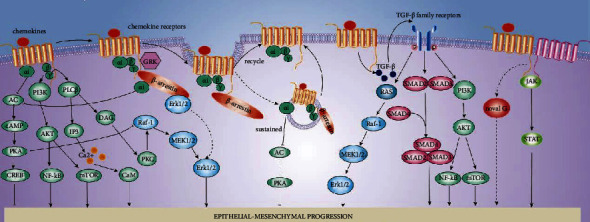
Signaling pathways to EMT initiated by chemokine receptors. Binding chemokines with their cognate receptors triggers G-protein signaling and JAK/STAT signaling at the very beginning if receptors are dimerized. Subsequently, *β*-arrestins bind to chemokine receptors, resulting in G*α*i-*β*-arrestin complexes and stimulation of G*α*i and Erk signaling. Most chemokine receptors lose their connection with *β*-arrestins after they are dragged into the cytoplasm and simply recycle back to the plasma membrane. During transactivation, chemokine receptors may stimulate receptor tyrosine kinases by elevating the expression of their ligands in the cytoplasm, such as TGF-*β*, EGF, and IGF.

EMT can be induced by several intracellular signaling pathways in neoplastic epithelial cells when they receive chemokine/GPCR signaling from the tumor microenvironment. Regarding to the signaling pathways that are triggered by chemokines, we need to refer to literature about GPCRs to deduce them. To date, as new biological techniques and research pop out, the modes of GPCR become a more complex picture [114]. Wang et al. proposed new insights into modes of GPCR activation, including biased, intracellular, dimerization, transactivation, and biphasic activation. These patterns are applicable in explaining the biological effects of chemokines [114]. Herein, we refer to these modes; categorize the signaling of chemokine receptors into G protein, *β*-arrestin, intracellular, dimerization transactivation, and MAPK signaling; and summarize each function in mediating EMT (Figure 2).

### 4.1. G-Protein Signaling

Most chemokine receptors have been identified to be G*α*i-dependent, since the first chemokine CXCL8 was found to mediate signaling via G*α*i subunit [115, 116]. G*α*i protein, a direct inhibitor of ACs, which subsequently suppresses cAMP and alters PKA and EPAC activation, has been shown to possess the ability to mediate the neoplastic transformation of normal cells when constitutively activated. In contrast, the G*α*s protein, a direct stimulator of ACs, has a tumor-suppressive role [117]. For example, conditional deletion of G*α*s results in the formation of basal-cell carcinoma by releasing PKA-mediated inhibition of sonic hedgehog (SHH) and YAP signaling pathways [118]. Moreover, Pattabiraman et al. uncovered a role of PKA in mesenchymal-to-epithelial transition and loss of tumor-initiating ability [119]. Notably, they used immortalized human mammary epithelial cells to model tumor-initiating cells in basal-like breast cancers rather than cancer cells [119]. This might explain why the in vitro model exhibited distinct c-EMT. These studies imply that G*α*i protein, which has an opposite effect to the G*α*s protein might be critical in maintaining the balance of epithelial and mesenchymal states. So far, few studies have directly elucidated the correlation between G*α*i protein and the EMT programme.

G*βγ* subunits, once thought to be only negative regulators of G*α*-dependent signaling, are now recognized to be more critical in GPCR signaling (reviewed in [120]), as well as chemokine receptors, because of their indispensability in chemotaxis [121]. In the early event, G*βγ* dimmers are required by many downstream effectors. Phospholipase C*β* (PLC*β*) is an effector directly activated by G*βγ* subunits. PLC*β* activation leads to the production of inositol-1,4,5-trisphosphate (Ins (1,4,5) P3) and diacylglycerol (DAG), which regulate the release of intracellular calcium (Ca2+) and activation of protein kinase C (PKC). The role of calcium flux in EMT was firstly described by Davis et al. EGF-mediated EMT was calcium-signal-dependent, partially owing to the effect of TRPM7 [122]. Reminiscent of the effect of G*α*q-coupled GPCRs, which can directly stimulate PLC*β* and induce calcium flux, Norgard et al. provided concrete evidence that G*α*q-coupled GPCRs mobilize calcium through Ca^2+^-calmodulin-Camk2b axis to assist cells in achieving a p-EMT state [123]. Interestingly, E-cadherin expression was not found to be downregulated in this study. Instead, it was translocated from the cell membrane to the cytoplasm [123]. However, the role of calcium signaling by G*βγ* subunits in mediating EMT remains to be explored. PI3K*γ*, a critical regulator of survival signaling, is also a direct effector of G*βγ* subunits [120]. Several studies have reported the participation of PI3K downstream chemokine receptors in mediating EMT. The CXCL12/CXCR4 axis mediates EMT via activation of PI3K-Akt/PKB in human sacral chondrosarcoma, breast cancer, and oral carcinoma cells [32, 50, 52]. Commonly, the PI3K-Akt signaling pathway works together with the MEK-MAPK pathway to induce EMT, and inhibition of either can only partially reverse the transition. In the context of CCR7, its ligand CCL19 was reported to upregulate Snail through MAPK and PI3K in gastric cancer, whereas CCL21 was reported to upregulate Slug through the same pathway in human chondrosarcoma [72, 78]. These studies highlighted G*βγ* subunits regulating MAPK and PI3K mediated EMT by chemokine receptor stimulation, and we will further discuss the role of MAPK signaling in the EMT programme in the latter part of this review.

### 4.2. *β*-Arrestin Signaling

Current studies on GPCRs indicate that *β*-arrestins not only can induce GPCRs internalization and desensitization but also can activate a novel pathway that is distinct from pathways induced by G proteins [124]. From the traditional point of view, most chemokine receptors hold the characteristic of inducing short and transient signals. They tend to rapidly terminate their signals and refresh themselves with the help of regulators of G-protein signaling (RGS) proteins, G-protein-coupled receptor serine/threonine kinases (GRKs), and *β*-arrestins [125, 126]. When chemokine receptors are internalized, they either become ubiquitinated or simply recycled to the membrane for resensitization. This hallmark of chemokine receptors has biological significance in sensing direction and chemotaxis [127, 128]. The internalization and desensitization processes are mainly regulated by GRKs and *β*-arrestins. Chemokine receptors phosphorylated by GRKs at multiple sites on the cytoplasmic COOH-terminus [126, 129] promote their binding affinity to *β*-arrestins. To date, *β*-arrestins have been found to serve as scaffold proteins that recruit several signaling molecules and emit signals parallel to G-protein-dependent signals [130]. For example, *β*-arrestins potently activate the MAPK kinase pathway by forming a CXCR4-*β*-arrestin complex that interacts with Raf [131]. Moreover, *β*-arrestin signaling may switch the coupling of GPCRs from canonical G*α* proteins to other G*α* proteins. For example, PKA-phosphorylated*β*2-adrenergic receptor switches G*α*s to G*α*i [132]. This switch of coupling G proteins can be well explained by recent research conducted by Smith et al. [133]. They discovered G*α*i:*β*-arrestin signaling complexes that formed downstream not only G*α*i-coupled GPCRs but also G*α*s- or G*α*q-coupled GPCRs at the plasma membrane. Moreover, the complexes are capable of forming scaffolds with ERK and promoting PTX-sensitive cell migration, which can also explain why G*α*i activation is necessary but probably not sufficient for chemotaxis [121, 134]. However, whether *β*-arrestin contributes to GPCR signaling remains controversial. O'Hayre et al. provided evidence that the loss of *β*-arrestins enhanced the potency and efficacy of isoproterenol- and epinephrine-induced ERK phosphorylation, suggesting that *β*-arrestins are not required for MAPK activation [135]. Notably, silencing *β*-arrestin-1 can reverse the EMT process initiated by the endothelin A receptor of ovarian cancer cells, while elevated *β*-arrestin-1 expression can promote EMT in prostate cancer cells via the Wnt/*β*-catenin pathway [136, 137]. *β*-Arrestin-1 has also been reported necessary in nicotine-induced EMT and protease-activated receptor 2 (PAR2) induced migration in lung cancer cells, with the involvement of Src kinase activity and MAPK signaling [138–140]. However, it still remains unclear whether or how *β*-arrestin downstream chemokine receptors play a role in the EMT programme. Since, even MAPK signaling, which is associated with various pathways, has been widely reported to be involved in EMT regulation. Further studies are required to identify novel mechanisms of dissection and phenotypic conformations.

### 4.3. Intracellular Signaling

Accumulated evidence indicates that some GPCRs can be activated not only on the cell surface but also inside the cell and trigger specific signals, suggesting that GPCR activation may be a result of intracellular events [114]. This phenomenon can be explained in two ways. GPCRs located in the cytoplasm can be activated intracellularly. And GPCRs located at the cell surface provide sustained signals after internalization. Chemokine receptors are primarily localized and activated at the cell surface. More importantly, they belong to the class A GPCRs, a group of GPCRs that form unstable endocytic complexes with *β*-arrestins. Chemokine receptors and *β*-arrestins disassociate rapidly after internalization and recycle back to the cell surface with MAPK activated in this transient process [141]. Together with G-protein signaling, we can conclude that signals induced by chemokine receptors from activation to resensitization are rapid and transient. Typically, the induction of EMT requires long-term cultivation (for days or weeks) and sustained signaling. This may explain why tyrosine kinase receptors are more popular at inducing EMT, since tyrosine kinase receptors seldom internalize and desensitize, thus providing stable signals to cells. Recent findings suggest that some GPCRs can (mostly class B GPCRs) mediate sustained G*α* signaling after internalization, such as vasopressin type 2 receptor (V2R) [142], parathyroid hormone receptor type 1 (PTHR) [143], TSH receptors (TSHRs) [144], and Sphingosine-1-phosphate (S1P) receptors [145]. One explanation for this sustained G*α* signaling is the recent discovery of an internalized GPCR-G protein:*β*-arrestin megaplex [146]. This megaplex architecture allows GPCRs to promote GDP-GTP exchange and G*α* activation, resulting in prolonged G protein signaling [146]. Few studies have reported sustained G protein signaling in chemokine receptors. Here, we use S1P receptors, a group of GPCRs that resemble chemokine receptors, as a reference. They belong to class A GPCRs, coupled to G*α*i protein, and regulate essential processes, such as adaptive immune cell trafficking, vascular development, and homeostasis [147]. However, as members of class A GPCRs, they demonstrate persistent signaling after the activation of FTY720P, a “functional antagonist,” by promoting the efficient internalization of S1P1 receptors. In conclusion, certain ligands of chemokine receptors might trigger long-lasting G*α*i signaling and bring massive influence on epithelial versus mesenchymal balance in cancer cells.

### 4.4. Dimerization Signaling

GPCRs were originally considered monomers. To date, much evidence has shown that these receptors exist as homodimers or heterodimers [148]. Surprisingly, these dimers can activate tyrosine kinase signaling pathways [149]. Using a sensitive bioluminescence resonance energy transfer system (BRET), Percherancier et al. found that CXCR4 exists as constitutive homo- and heterodimers [150]. Chemokine receptors can form dimers with different receptors. For example, CXCR4 forms heterodimers with CXCR7 [151], CCR5 [152], CD4 [153], CCR2 [154], and even *δ*-opioid receptor [155]. Heterodimers may conduct a *β*-arrestin switching or G protein switching. For instance, CXCR4-CXCR7 heterodimers block G*α*i activation and induce *β*-arrestin signaling pathway [156], and costimulation of CCR2-CCR5 with their cognate ligands leads to novel G*α*q/11 signaling, instead of the classical G*α*i signaling [157]. Changes in the balance of homo- and heterodimers, as well as the formation of novel heterodimers, could have significant effects on signaling [124], and whether there is a typical heterodimer in cancer that exclusively manages the EMT programme requires further discussion.

### 4.5. Transactivation Signaling

Transactivation means several GPCR agonists induce rapid tyrosine phosphorylation and activation of receptor tyrosine kinases. Currently, two mechanisms of RTKs and GPCRs interaction have been identified as “ligand-dependent” and “ligand-independent” mechanisms. A typical example of ligand-dependent mechanisms is GPCRs that activate EGFR. GPCRs first activate the Src kinase. Activated Src then elevates the expression of matrix metalloproteinases (MMPs), which shed and cleave HB-EGF, a classical endogenous ligand for EGFR, from the membrane and subsequently captured by EGFR [114]. In the context of chemokine receptors, the CXCL12-CXCR4 axis mediates TGF-*β* formation and activates TGF-*β* signaling, which in turn mediates CXCL12 formation through the pSmad2/3 pathway. Together, CXCL12 and TGF-*β* form autocrine signaling loops in myofibroblasts of invasive human breast cancers [158]. In a ligand-independent mechanism, GPCRs can transactivate EGFR by forming GPCR-EGFR heterodimers or complexes (reviewed in [114]). Similarly, CXCR4 transactivated by insulin-like growth factor receptor (IGF-1R) and S1P1 receptor by platelet-derived growth factor (PDGF) are both activated by forming GPCR-RTK complexes [159, 160]. As mentioned above, RTKs can be important regulators of the EMT programme. For example, TGF-*β* can induce EMT through the RAS-RAF-MEK-MAPK pathway or SMAD2-SMAD4-SMAD3 complexes [10]. Thus, the induction of EMT may be a joint effort by both chemokine receptors and RTKs.

### 4.6. MAPK Signaling

MAPK is phosphorylated by multiple signaling induced by chemokines. In the context of G protein signaling, the G*α*i subunits activate MAPK through the cAMP-PKA-cRaf pathway and Rap1-cRaf pathway. *βγ* subunits activate MAPK through PLC*β*/PI3K-Src-Shc-Sos pathway (reviewed in [161]). In the case of the *β*-arrestin pathway, *β*-arrestin not only functions as scaffolds to enhance cRaf-1 and MEK-dependent activation of MAPK [131, 162] but also forms G*α*i:*β*-arrestin complexes to activate MAPK [133]. However, *β*-arrestin-mediated Erk MAPK activation and MAPK regulation EMT are all required further mechanistic and phenotypic studies. Studies have found biphasic activation of GPCRs. AT1R-mediated MAPK phosphorylation has two peaks. The first peak is transient and appears at 2 min after activation, while the last peak is sustained and appears at 120–150 min. The appearance of the first peak might be a result of G*α*:*β*-arrestin signaling complexes, while the last might be stimulated by G*βγ* subunits or from intracellular signaling. The inhibitors of MAPK might inhibit all the sources of MAPK phosphorylation, which lead us to miss the effect of *β*-arrestins.

Above all, multiple chemokine/GPCR signaling pathways have been proven correlated with EMT. Among them, the MAPK and PI3K signaling from G*βγ* subunits, JAK/STAT signaling form dimerization signaling, and crosstalk between chemokine receptor and Wnt signaling are well focused. And their inhibitors have been proven to reverse or inhibit the EMT process. However, signaling the effect of G*α*i and *β*-arrestin has long been ignored. Indeed, we believe EMT is a result of complicated signaling networks. Further study should complete these networks and distinguish them from each other if possible.

## 5. Conclusion and Perspective

The chemokine family is one of the pivotal regulators of EMT in the tumor microenvironment with an array of signaling pathways that can activate the EMT programme. However, the limited pathway has been proven to be dominating in the EMT programme and available in all types of cancer cells. Inspired by calcium-mediated p-EMT [123], we can deduce that different signals emitted by chemokine receptors may activate different EMT programmes. Our knowledge of chemokine-triggered signaling and the EMT programme has advanced, but still little is known about how different chemokines and signaling pathways work in combination to drive EMT in different tumors. Chemokines play a pivotal role in metastatic dissemination due to their functions in directing cell migration, facilitating transendothelial migration, mediating cell adhesion, and recruiting stromal and immune cells. Uncovering how cancer cells “hijack” chemokine receptors to mediate cell migration highways will be an exciting and promising direction.

EMT and EMT-TFs are associated with metastasis. However, whether the EMT programme is critically involved in metastasis or merely casual requires concrete experimental evidence. EMT-TFs have a common function in the activation of the EMT programme with each having non-redundant functions [163]. Therefore, the appearance of the EMT phenotype in cancer indicates the activation of certain EMT-TFs. For example, Snail2 was detected in p-EMT malignant cells at the leading edge of cancer [12]. To date, EMT-TFs have been proven to play a critical role in metastasis with a tissue-specific property and spatiotemporal expression patterns [3, 164]. However, the EMT programme has long been associated with the metastasis process, and EMT-TFs functions should not be simply extrapolated as functions of the EMT programme.

EMT programmes have been shown strongly associated with the processes of primary tumor invasion and dissemination, which are considered the first step in the metastatic cascade. Without dissemination, metastases do not develop, but highly invasive cancer cells also need epithelial characteristics to settle and resume tumor growth at metastatic sites. Thus, EMT programmes might not always positively contribute to the whole process of complex cancer metastasis. For example, EMT programme-targeted therapy may activate the MET programme, which encourages colonization of cancer cells [164]. In addition, the transiency of EMT-TFs and effectors, abundance of EMT receptors, and redundant function of EMT pathways make EMT difficult to target experimentally and clinically.

**Figure 3:**
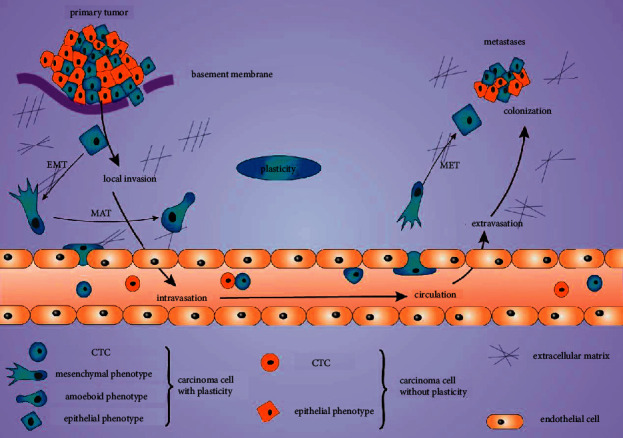
Depiction of epithelial-mesenchymal plasticity in metastasis. Carcinoma cells with plasticity or without plasticity were primarily outlined by a basement membrane. Carcinoma cells with plasticity undergo EMT or MAT, resulting in local invasion, intravasation, circulation, extravasation, and colonization. In this process, carcinoma cells with plasticity decide which morphology (mesenchymal or amoeboid) to transform in response to their circumstances. As carcinoma cells colonize, EMT cells may transform back to epithelial phenotype by MET to proliferate. Abbreviations: EMT, epithelial-mesenchymal transition; MAT, mesenchymal-amoeboid transition; and MET, mesenchymal-epithelial transition.

Notably, only a few cancer cells could undergo complex processes of metastasis, including local invasion, dissemination, intravasation, transportation through circulation, extravasation, and final colonization. Interestingly, those cells must go through not only the EMT process during dissemination at the very beginning of metastasis but also the MET process at final colonization [3], suggesting that cancer cells could not be in a certain consistent statements to achieve final metastasis. The key abilities include switching statements from one to another, such as EMT or MET, which as known as “plasticity,” one of the hallmark features of cancer cells [165]. Therefore, plasticity might be a more constant property of metastatic cancer cells, and targeting plasticity might be a suitable strategy for cancer metastasis therapy (Figure 3).

## Figures and Tables

**Table 1:** Summary of literature regarding chemokine-mediated EMT.

Chemokine-receptoraxis	Category	Cancer type	Marker used		Signaling pathway		Method	Refs
CXCL1-CXCR1	Inflammatory	Glioma	N-cadherin, vimentin		NF-*κ*B/Snail		WB, IF	[22]
CXCL1-CXCR1/CXCR2		Prostate cancer	E-cadherin		Src pathway		WB	[23]
		Ovarian cancer	E-cadherin, vimentin		Wnt/*β*/catenin		WB	[24]
CXCL2-CXCR2		Colon cancer	EpCAM, Snail, Twist, vimentin		G*α*i-2 and G*α*q/11 pathway		WB	[25]
CXCR2		Breast cancer	E-cadherin		P85*α*/Akt1		WB	[26]
CXCL5-CXCR2		Colorectal cancer	E-cadherin, ZO-1 N-cadherin, vimentin		MAPK/Elk-1/Snail		WB, IF	[27]
		Papillary thyroid cancer	E-cadherin, N-cadherin, vimentin		*β*-catenin signaling		WB, IF	[28]
		Head and neck cancer	E-cadherin, vimentin, Snail		MAPK/GSK-3*β*/Snail		Morphology, WB, qPCR	[29]
		Hepatocellular cancer	E-cadherin, vimentin		PI3K/Akt/GSK-3*β*/Snail		Morphology, IF, WB, IHC	[30]
CXCR4/CXCR2		Gastric cancer	E-cadherin, N-cadherin, vimentin, Snail		CXCR2/NF-*κ*B/*p*65-CXCR4 CXCR4/JAK/STAT3/CXCR2		WB	[31]
		Breast cancer	ZEB1, cadherin 11, E-cadherin		PI3K/Akt/mTOR Raf/MEK/MAPK		WB	[32]
CXCL8-CXCR1/CXCR2		Breast cancer	E-cadherin, N-cadherin, vimentin, MMP9		Unreported		qPCR	[33]
		Colon cancer	E-cadherin, N-cadherin, vimentin, *α*-SMA		PI3K/Akt/NF-*κ*B		WB, qPCR	[34]
		Thyroid cancer	Zeb, Slug, Snail		Akt/Slug		Morphology, WB	[35]
		Triple-negative breast cancer	E-cadherin. MMP2		PI3K/Akt		WB	[36]
CXCL9-CXCR3		Head and neck cancer	E-cadherin, vimentin		Akt pathway		IF, WB	[37]
CXCL10-CXCR3		Colon cancer	E-cadherin, ZO-1, cytokeratin, occludin, desmoplakin, fibronectin, N-cadherin, vimentin, Snail		PI3K/Akt/GSK-3*β*/Snail		Morphology, qPCR, IF, WB	[38]

CXCL12-CXCR4	Homeostatic	Colorectal cancer	E-cadherin, Snail, vimentin, MMP7		CXCR4/miR-133a/RhoA Wnt/*β*-catenin		IHC, WB	[39, 40]
		Papillary thyroid cancer	E-cadherin, N-cadherin, vimentin		NF-*κ*B pathway		Morphology, WB, IF	[41]
		Gastric cancer	ZEB1/2, Twist2, Snail, E-cadherin, vimentin		STAT3-ZEB1 and Cav-1-c-EMT		Morphology, WB, IF	[42, 43]
		Hepatocellular cancer	E-cadherin, N-cadherin, vimentin		Wnt-*β*-catenin		IHC, WB	[40, 44]
		Ovarian cancer	E-cadherin, N-cadherin		Wnt-*β*-catenin		WB	[45]
		Non-small-cell lung cancer	E-cadherin		Akt pathway or MAPK pathway		IHC	[46]
		Pancreatic cancer	E-cadherin, vimentin		Non-canonical hedgehog pathway (SDF-1/CXCR4/SMO)		WB	[47]
		Lung cancer	E-cadherin, vimentin N-cadherin, Slug		CXCR4/*β*-catenin/PPAR*δ*		WB, IF	[48]
		Head and neck cancer	E-cadherin, keratin, N-cadherin, vimentin, MMP2, MMP9			PI3K/Akt	WB, IHC, IF, qPCR, morphology	[49–51]
		Sacral chondrosarcoma	Snail, N-cadherin	MEK/MAPK and PI3K/Akt			WB	[52]

CXCL13-CXCR5	Homeostatic	Breast cancer	E-cadherin, N-cadherin, vimentin, Slug, Snail	Src/PI3K			WB, qPCR, IF, morphology	[53]
		Renal cell carcinoma	E-cadherin, N-cadherin, vimentin	Akt pathway			WB	[54]

CXCL16-CXCR6	Homeostatic	Gastric cancer	E-cadherin, ZO-1N-cadherin, Snail, Slug	Akt pathway MAPK pathway			WB, IF, morphology	[55, 56]

CCL2-CCR2	Inflammatory	Hepatocellular cancer	E-cadherin, vimentin, Snail	Hedgehog pathway (CCR2/SMO/Gli-1)			WB, qPCR	[57, 58]
		Gastric cancer	E-cadherin, vimentin, ZEB2	CCR2/MAPK/ELK1/EGR1/ZEB2			IF, WB, qPCR	[59]
		Breast cancer	E-cadherin, vimentin, fibrinogen	MAPK/GSK-3*β*/Snail			Morphology, WB, IF	[60]
		Prostate cancer	E-cadherin, Snail, MMP9	CCR2-STAT3			WB	[61]
		Head and neck cancer	E-cadherin, Snail, vimentin	Unreported			WB, IF	[62]

CCR4	Dual	Hepatocellular cancer	E-cadherin, N-cadherin, vimentin, Slug, Snail	MAPK-akt-MMP2			WB, IF	[63]
CCL17-CCR4		Hepatocellular cancer	E-cadherin, N-cadherin vimentin, Snail	Wnt/*β*-catenin			WB	[64]
CCR5	Inflammatory	Melanoma	E-cadherin, N-cadherin, vimentin, Snail, Slug	PI3K/AKT/GSK3*β*			WB, IF, IHC	[65]
CCL18-CCR6		Pancreatic ductal adenocarcinoma	Snail, E-cadherin	Unreported			qPCR, WB	[66]
CCL20-CCR6		Colon cancer	E-cadherin, vimentin, N-cadherin, Snail1, ZEB1, *β*-catenin, *α*-SMA	Unreported			WB, qPCR	[67]
		Gastric cancer	N-cadherin, vimentin, MMP2	CCR6/CrkL/MAPK1/2			WB	[68]
		Breast cancer	E-cadherin, ZO-1, N-cadherin, vimentin, Snail	NF-*κ*b/Snail, mTOR/S6 kinase, PKC*δ* ccr6/VEGF			WB, qPCR	[69]
CCL20-CCR6		Head and neck cancer	E-cadherin, vimentin	Unreported			WB, qPCR	[70]

CCL19-CCR7	Homeostatic	Breast cancer	E-cadherin, N-cadherin, vimentin	Akt pathway			WB	[71]
		Gastric cancer	Snail, E-cadherin, MMP9	MAPK/Snail and PI3K/Snail			WB	[72]
		Ovarian cancer	E-cadherin, N-cadherin, Snail, MMP9	CCR7-CrkL-MAPK			WB	[73]
CCL21-CCR7		Head and neck cancer	E-cadherin, N-cadherin, vimentin	JAK/STAT			WB, IF, morphology	[74]
		Pancreatic cancer	E-cadherin, N-cadherin, LYVE-1	MAPK/NF-*κ*B			WB	[75]
		Lung cancer	E-cadherin, vimentin, Slug	MAPK pathway			WB, qPCR	[76]
		Breast carcinoma	E-cadherin, vimentin, N-cadherin, Slug	Unreported			WB, qPCR	[77]
		Chondrosarcoma	E-cadherin, N-cadherin, Slug	MAPK/Slug and PI3K/Akt/Slug			WB	[78]
CCL18-CCR8		Bladder cancer	E-cadherin, MMP2	MMP2 pathway			WB	[79]
CCR9		Osteosarcoma	E-cadherin, N-cadherin, vimentin, Twist, Snail, MMP-1	Wnt/*β*-catenin			WB qPCR	[80]
CCL28-CCR10		Head and neck cancer	E-cadherin, *β*-catenin, Slug, Twist1, Snail	Inhibit EMT CCR10/RAR*β*			WB, IF	[81]

XCL1-XCR1	Dual	Breast cancer	E-cadherin, N-cadherin, vimentin	MAPK/HIF-1*α*			WB	[82]
